# Can Ingestion of Lead Shot and Poisons Change Population Trends of Three European Birds: Grey Partridge, Common Buzzard, and Red Kite?

**DOI:** 10.1371/journal.pone.0147189

**Published:** 2016-01-22

**Authors:** Carolyn B. Meyer, Joseph S. Meyer, Alex B. Francisco, Jennifer Holder, Frederik Verdonck

**Affiliations:** 1 Arcadis, Lakewood, Colorado, United States of America; 2 Department of Chemistry and Geochemistry, Colorado School of Mines, Golden, Colorado, United States of America; 3 Arcadis, Walnut Creek, California, United States of America; 4 ERM, Carpinteria, California, United States of America; 5 ARCHE, Gent, Belgium; Hungarian Academy of Sciences, HUNGARY

## Abstract

Little is known about the magnitude of the effects of lead shot ingestion alone or combined with poisons (e.g., in bait or seeds/granules containing pesticides) on population size, growth, and extinction of non-waterbird avian species that ingest these substances. We used population models to create example scenarios demonstrating how changes in these parameters might affect three susceptible species: grey partridge (*Perdix perdix*), common buzzard (*Buteo buteo*), and red kite (*Milvus milvus*). We added or subtracted estimates of mortality due to lead shot ingestion (4–16% of mortality, depending on species) and poisons (4–46% of mortality) reported in the UK or France to observed mortality of studied populations after models were calibrated to observed population trends. Observed trends were decreasing for partridge (in continental Europe), stable for buzzard (in Germany), and increasing for red kite (in Wales). Although lead shot ingestion and poison at modeled levels did not change the trend direction for the three species, they reduced population size and slowed population growth. Lead shot ingestion at modeled rates reduced population size of partridges by 10%, and when combined with bait and pesticide poisons, by 18%. For buzzards, decrease in mean population size by lead shot and poisons combined was much smaller (≤ 1%). The red kite population has been recovering; however, modeled lead shot ingestion reduced its annual growth rate from 6.5% to 4%, slowing recovery. If mortality from poisoned baits could be removed, the kite population could potentially increase at a rapid annual rate of 12%. The effects are somewhat higher if ingestion of these substances additionally causes sublethal reproductive impairment. These results have uncertainty but suggest that declining or recovering populations are most sensitive to lead shot or poison ingestion, and removal of poisoned baits can have a positive impact on recovering raptor populations that frequently feed on carrion.

## Introduction

Effects of ingestion of lead shot by waterfowl and wetland birds has been studied at the population scale as well as the individual scale [1–3]. However, few studies exist about the effects of lead shot ingestion on population trends of non-waterbird avian species, despite the increasing amount of literature on individual effects. Major literature reviews of lead shot ingestion [4–7] collectively show that individual terrestrial birds, and even large numbers of birds in some isolated events, can be adversely affected. However, little is known about lead shot toxicity on terrestrial bird population growth rates or probability of extinction, or how lead shot can interact with other chemical stressors at the population level. In particular, poison-soaked baits often kill terrestrial carnivorous birds feeding on the bait and reduce population growth [8]. These poisons combined with lead shot ingestion may have large effects on population growth or change the direction of population trends.

This study demonstrates use of a population model to address the question of how lead shot ingestion might be changing population sizes of three terrestrial bird species that are susceptible to lead shot as well as effects of a second class of chemical stressors in European countries, poison. Birds can be poisoned through pesticide poisons ingested as granules or on coated seeds or by ingesting baits laced with poison. Mortality from ingestion of lead shot is a small proportion of total mortality (see Discussion section), but the combination of lead shot poisoning and poisoning from pesticides or baits might increase extinction probabilities for some species. This study models the effect of removing mortality from both types of chemicals. That is, we address an important question: In combination, are ingested lead shot and poison substantially reducing population sizes over time and affecting their sustainability? Answering such a question requires appropriate and reliable data as input for the models, and thus our analysis also identifies data gaps needed to be filled to improve model results in the future.

Poison is narrowly defined in this paper as chemicals that are designed to kill target species and includes crop protection products (pesticides), rodenticides, and lethal chemicals applied to baits to control predators. Non-target species might ingest these poisons, resulting in secondary poisoning. Poison applied to baits that are intended to target raptors are illegal in Europe but still occur and are thus included in the category of poisons.

Population-level changes were evaluated by (1) estimating the proportion of total mortality attributable to lead shot ingestion or poisoning using empirical mortality data from field studies in the United Kingdom (UK) or France and (2) comparing change in population size and extinction risk with and without such mortality using population viability analysis models. This approach is often taken to assess effects of stressors on wildlife population trends [8–10]. Data from laboratory toxicity studies are less useful than empirical mortality data because laboratory studies cannot provide exposure or annual mortality rates of lead shot or poison ingestion for populations; instead, they only provide mortality rates of individuals exposed to chemicals at laboratory-selected intensities, durations, and frequencies. Moreover, for lead shot, mortality rates in the laboratory vary greatly due to differences in species, diet, grit consumed, gastrointestinal passage rates, and size of shot [11–13]. Therefore, reported mortality rates from these chemical stressors from field studies are the most useful and most-integrative data for modeling potential responses of real-world populations.

The objective of this study is to estimate and bound the possible effects of lead shot and secondary or illegal poisoning on three populations of common European terrestrial bird species that are susceptible to these substances: grey partridge (*Perdix perdix*), common buzzard (*Buteo buteo*), and red kite (*Milvus milvus*). We estimate the change in population size of these three species when exposed to lead shot in small-game hunting areas in various locations in Europe. Data for estimating population changes came from the UK for the kite and from Germany and the UK for the buzzard. Data for the partridge came from continental Europe and the UK. This study does not focus on localized shooting ranges, where impacts from lead shot ingestion might be larger; instead, it focuses on lead shot in non-wetland soil of the more ubiquitous small-game hunting areas, and then evaluates the effect of poisons in addition to lead shot ingestion in such areas.

Less is known about adverse reproductive effects from lead shot and poison ingestion than effects on survival. Typically, fecundity effects have been ignored in population modeling of lead shot ingestion or poisoning [1–2, 8–9]. We included potential reproductive effects in a sensitivity analysis to test how changes in fecundity might change the results. Additionally, modeling exercises to evaluate stressor effects are often criticized if they do not model actual trends observed in populations. Therefore, to improve realism, the baseline models representing actual conditions were developed and calibrated against observed trends of actual populations, before adjusting for effects of lead shot and poison ingestion. In that context, our approach is a retrospective-scenario analysis, adding or removing stressors to population dynamics observed over a specified period to evaluate how trends might change. The scenarios produce hypothetical (“what if”) results because of inevitable uncertainty in the model parameters (see later sections). Because of such uncertainty, the emphasis of this paper is to show how population models can be used to evaluate effects of multiple chemical stressors on vertebrate populations, and the type of data required for such models.

## Methods

Because effects of lead shot ingestion on population growth of terrestrial birds have not been modeled before, the focus of the analysis was first on lead shot effects and secondly on additional effects from other poisons. The three species selected for this analysis had to meet the following criteria. They must be a European bird that is representative of one of the two guilds of terrestrial birds that are most likely to ingest lead shot: (1) granivorous birds that obtain lead shot while foraging for seeds or grit and (2) carnivorous/scavenger birds that obtain lead shot mostly from ingesting tissue of crippled hunted prey or carrion [5]. The three species should have both lead shot exposure data and long-term population dynamics data available in the literature. Finally, the selected populations of the three species together should represent increasing, declining, or stable trends in population size, because poisoning has different effects on the population, depending on which trend is represented. In Europe, the grey partridge, common buzzard, and red kite meet these criteria.

The grey partridge population that we chose to model is the continental European population, which was stable in the early 20^th^ century but has declined since the 1970s [14] and is exposed to toxicants such as pesticides and lead shot remaining in fields after hunting. This population is a mixture of released and wild populations, having been manipulated by gamekeepers for over a century [15]; and the modeling results apply to this mixed population. The common buzzard generally has stable population trends in most of the European Union (EU) [16], and we focused on the wild, stable population studied in Germany. The red kite population trends are variable in Europe, but we selected a wild population in Wales of the UK where the red kite is increasing as it recovers from a severe decline, and where it is a year-round resident [17]. Year-round residency is important because migratory red kites elsewhere in the EU leave their breeding range before the hunting season and are less exposed to lead shot ingestion.

Frequent feeders of carrion, such as the red kite [8], are more exposed to poisoned bait and to lead in unretrieved carcasses that were shot by hunters than are raptors that often hunt their prey, such as the common buzzard [18]. The red kite has the highest lead shot exposure rates reported to date for European raptors (higher even than vultures [19]) and thus are a good model species to evaluate. Common buzzards, though less exposed because they more frequently hunt their prey, are of interest because they are a well-studied common raptor that also feeds on carrion. Buzzards have exposure to lead shot and poisons year-round throughout the EU because they are at most a short-distance migrant; however, effects of lead shot and poisons on population trends for such common species are unknown.

### Percentage of annual mortality from lead shot and poisons

#### Lead shot ingestion

Models of the probability of a granivorous bird ingesting lead shot are available [7] but do not predict the annual bird mortality expected from lead shot ingestion. To approximate annual mortality from lead shot uptake, we employed the approach used by wildlife biologists of estimating percentage of total annual mortality due to lead shot ingestion, poison, and other causes of mortality, using data from pathology and radio-telemetry studies conducted in the EU [20–25]. In the studies we used, the diagnosis of toxicosis from lead shot for partridges was based on (1) extreme emaciation combined with a characteristic green staining of the gizzard and gut contents due to excess bile, (2) necrosis of the gizzard lining and (3) marked anemia [20]. The diagnosis for red kites was a pathologist’s weight of evidence approach after eliminating other potential causes, including examining carcasses for (1) elevated lead concentration in liver (> 15 mg/kg dry weight [dw]), (2) dilated gall bladder, (3) high lead concentrations in bone (> 20 mg/kg dw), (4) lead shot in mouth, and (5) lead isotope ratios in liver and bone compared to lead isotope ratios in gunshot [21]. Though buzzard tissue often contains elevated concentrations of lead [22], we could not find studies that reported lead shot ingestion as a suspected cause of death of common buzzards.

The officially-listed causes of death from wildlife studies are the proximal, direct causes of death that probably underestimate the frequency of lead-caused deaths. Lead ingestion likely increases susceptibility of birds to other causes of death and may be the ultimate, underlying cause of some deaths. For example, sublethal lead poisoning may not be fatal but could impair the immune system, increasing susceptibility to disease or increasing inattentiveness, which in turn increases susceptibility to accidents and predation that are reported as proximal causes of death. Also, it is possible that common buzzards have died of lead toxicosis, but pathologists did not report it as such because they could not easily identify it as a cause. Therefore, we obtained not only the reported proportions of cause of death but also the proportion of deaths that might have been ultimately caused by lead shot ingestion based on observed percentage of dead birds with (1) elevated, subclinical lead concentrations (≥ 6.5 mg/kg dw in liver [21–22]) in tissue for raptors (even when reported as dying from another cause) and (2) emaciated condition for partridges (called “bad” body fat in [23]), a common symptom of lead exposure for partridges (note: percentage of dead birds with elevated lead in tissues was unavailable for partridge). These percentages of ultimate cause of death are probably overestimates because subclinical lead concentrations do not always lead to death, and low body fat can be from other causes (e.g., from starvation and disease). Therefore, we used the ultimate-cause estimates as an upper bound of the percentage of annual mortality from lead shot ingestion and the reported proximal estimates as a lower bound; and we assume the midpoint values may be more-realistic estimates.

**Fig 1:**
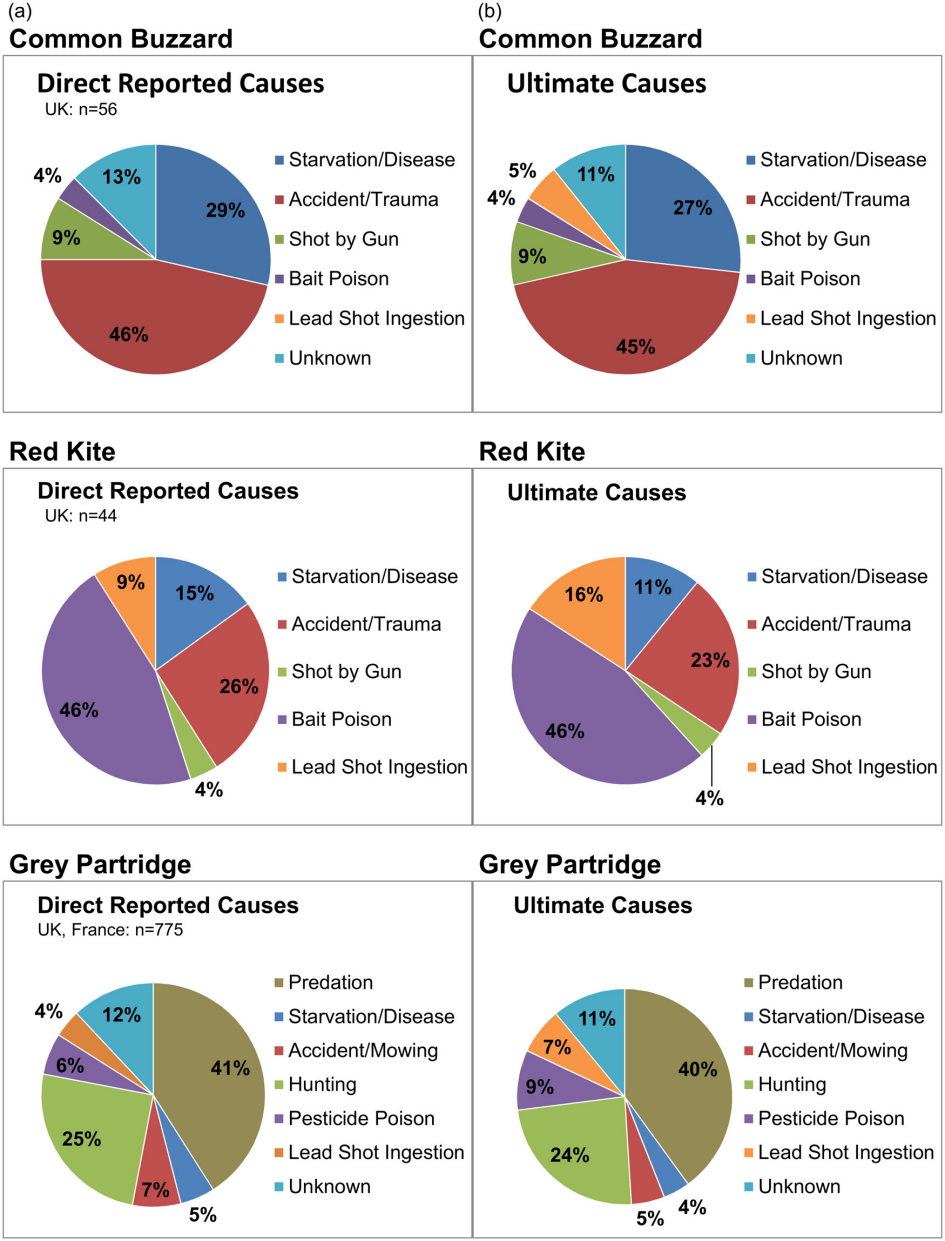
Estimated percentages of mortality attributable to various causes (n = number of birds) [10, 20–23, 25]. Direct reported causes are proximal causes of death and represent lower-bound estimates of contribution of ingestion of lead shot and poison to mortality in selected study areas used in the modeling exercise (a, left panel). Possible ultimate causes by lead shot or poisoning (see text) were added to the reported percentages to represent upper-bound estimates (b, right panel).

We estimated percentage of total mortality from different causes based on mortality studies of the three species in areas in the EU where lead shot ingestion was high and poison prevalent (Fig 1). Percentage of deaths from lead shot ingestion for the common buzzard, red kite, and grey partridge were modeled as 0, 9, and 4%, respectively for direct proximal cause of death. The data sources for these estimates of mortality caused by lead shot and other causes were: a wild common buzzard population in the UK [22], a reintroduced red kite population in the UK [21], the partridge population in continental Europe for hunting mortality [10], and a mix of wild and released partridges for non-hunting mortality in the UK and France [20, 23, 25]. For potential ultimate cause of death, the percentages were modeled as 5, 16, and 7% for the buzzard, kite, and partridge, respectively (using raptor elevated subclinical [≥ 6.5 mg/kg dw] liver lead concentration and partridge “bad” body fat data [21–23], Fig 1). Percentages of mortality from different causes appear to be relatively similar whether from wild or introduced populations [22, 25, 26, 27]. These percentages, however, are approximate estimates (e.g., partridge estimate is from a composite of studies) and exact percentages, particularly from poison and lead shot, will vary from population to population and with biases caused by the carcass-collection method. However, they meet this paper’s simple purpose of illustrating how lead shot ingestion at these levels might change population trajectories through time. The results are not meant to be a definitive description of lead shot and poison ingestion effects on bird populations in Europe. Reliable data on annual mortality from lead shot and poison ingestion and fecundity effects in different study areas, as well as metapopulation modeling across patchy habitats, will be needed to improve the realism and applicability of these models across Europe in the future.

For the partridge, the estimated mortality percentages from lead shot ingestion were applied to chicks (≤ 6 weeks old) in the spring and adults in the autumn/winter period when most die from lead shot ingestion [6]. In this model juveniles > 6 weeks old are included in the adult class. Chick mortality was included for the partridge because Potts [20] reported that chicks ingest lead shot while foraging. Chick mortality was not included in the standard model runs for raptor nestlings because of low availability of shot game in the breeding season, and no studies to date clearly demonstrate impacts of lead shot on raptor fecundity. Therefore, fecundity, defined as female raptor fledglings or female 6-week old partridge chicks produced per adult female, was reduced by lead shot ingestion in the partridge model but not in the raptor models. However, fecundity was addressed further in the sensitivity analysis of raptor and partridge models to capture the uncertainty in hatching effects and raptor nestling exposure, as discussed in the *Sensitivity Analysis of Fecundity Effects* section.

#### Poisoning

To estimate mortality from poison, we used an approach similar to our approach for lead shot. We included poison as the ultimate cause of death if a study reported lethal concentrations of the poisonous chemical in tissues, even if the necropsy concluded that a bird died of a different non-chemical cause (which occurred with the partridge but not with the raptors). For example, in Bro et al. [23], 6% of grey partridges were reported as dying from pesticides. However, an additional 3% died from predation even though they had lethal concentrations of pesticides in their tissue, thus leading to our classification of 9% assumed to ultimately have died from poison. We did not model indirect effects of poisons on food, such as pesticides reducing insect prey for partridge chicks, which has already been documented to reduce partridge populations and its effect has been modeled [24]. This paper focuses on the toxic action of directly poisoning the birds.

We modeled percentage of deaths reported to be caused directly by illegal and secondary poisoning for the common buzzard, red kite, and grey partridge as 4, 46, and 6%, respectively (Fig 1). Adding potential ultimate causes of secondary poisoning from pesticides on mortality increased that estimate for partridge to 9%. The “unknown” category was included in mortality percentages (Fig 1) only if the author of the source data included it when calculating such percentages. For upper-bound estimates, we partitioned some of the unknown category into lead shot ingestion or poisoning based on the number of those unknowns with high tissue concentrations of lead [22] or pesticide [23].

Consistent with other published raptor population models on poisoning [8, 28], the poisoning percentages were not applied to raptor nestlings because predominantly adults are exposed to poisoned bait [29]. When raptor chicks died of poisoning, one or both parents usually also died [29]; and the model already accounts for reproductive loss when parents die. Similarly, the poisoning percentages were not applied to partridge chicks, although they were applied to first year and older birds after they were > 6 weeks old. Partridge chicks eat mostly invertebrates [24] and likely much less of the pesticide-coated seeds (or granules) than older birds. We could not find studies that report chicks dying directly of pesticide poisoning. However, given uncertainty about the effects of poisons on partridge or raptor chicks, we included impairment of fecundity in the sensitivity analysis, allowing assessment of how much a reduction of fecundity might change the outcomes of the models.

### Sensitivity of model output to reduced fecundity

In an analysis of the sensitivity of the population models to changes in fecundity, we modeled lead shot ingestion or poisons as reducing hatching success of the partridge by 0, 5, and 10%. This range was evaluated because one laboratory study demonstrated no net effect on number of normal bobwhite quail chicks (*Colinus virginianus*) hatched per hen from ingestion of 15 to 100 lead shot by the hen (i.e., it showed reduced hatchability but increased egg production [13]), and another demonstrated 20% reduction in hatching of eggs laid by mourning dove hens that ingested 1 lead shot (but with no effect on egg production [11]). These laboratory results are for individual birds and do not account for the proportion of hens in a population ingesting lead shot within the months prior to laying eggs (often greater than a month is needed for complete excretion of lead from bobwhite quail [30]). It also does not account for the lower availability of shot in spring after burial of the lead shot by cultivation [6]. Nonetheless, to evaluate the importance of a range of possible fecundity effects from lead or any poison not accounted for in the standard model runs (e.g., see [31]), we created scenarios of soil lead shot and/or pesticides reducing breeding success of every hen in a population by 5% and then by 10%, which is equivalent to half the hens experiencing 10% or 20% reduction in hatching success.

We also included a 0 and 5% reduction in fecundity in the sensitivity analysis for the two raptor species. We are unaware of any studies that have demonstrated net sublethal adverse effects of lead shot ingestion or bait poisons on raptor fecundity in the field (e.g., [28–29,32]), or toxic concentrations of lead in raptor nestling blood [33–36]. However, in some areas, parent birds may feed spring/summer-poached or legally-shot prey to nestlings (where nestlings die but parents survive). Also, lead shot ingestion in female adults might reduce clutch size or hatching success. Such reductions could decrease fecundity somewhat (likely < 5% due to low availability of shot prey) if not compensated by increased survival in the juveniles from reduced competition (but such compensation was reported in [19]).

### Population Models

To evaluate changes in the population size, growth, and extinction probability from ingestion of lead shot and poison, we used the more realistic version of population models that incorporates density-dependence and stochasticity. Density dependence is needed to correctly model population growth, recovery rates and changing equilibrium (steady-state) population sizes of these species. Stochasticity is needed to estimate extinction or quasi-extinction probabilities. Quasi-extinction is the probability of a population size declining below a specified threshold of concern greater than zero. We focus on probabilities of quasi-extinction rather than ultimate extinction (i.e., when no individuals remain) because of phenomena that act on very small population sizes such as inbreeding, the Allee effect, and demographic stochasticity [37], which our models do not take into account [10]. Therefore, our estimates of extinction probability are higher than estimates of probability of ultimate extinction. We compared the output of these models to the outputs of the models that included and excluded the contribution of lead or poison to mortality.

#### Grey partridge model

For the grey partridge, we first ran the stochastic, demographic population model published by De Leo et al. [10] for the continental population of grey partridges in Europe (equations in their appendix and S1 Fig). This partridge population was at a relatively high steady-state density in the 1930s to 1960s and then decreased to a lower steady-state density until 1995, when it appeared to decrease again to an even lower steady state [10, 14, 38]. De Leo et al. [10] parameterized their model and density-dependent relationships for spring population growth rates and autumn/winter mortality using empirical data for the middle period of 1965 to 1993 [S1 Fig]. Those empirical data were from continental Europe during a period when lead shot ingestion was high compared to earlier in the century [20]. We modeled lead shot and poison effects using their model during this middle period. The model begins with the estimated population density each year, which is changed by spring growth rate and mortality rates randomly selected from lognormal distributions for each year (where the survival rate is constrained to not exceed 1). Specifically, early-spring partridge density is multiplied by the density-dependent per capita rate of increase in spring; then a constant hunting mortality rate is subtracted, followed by application of a density-dependent autumn-winter survival rate to predict the population density at the beginning of the following year. The environmental variation is modeled with the standard deviations of spring growth rate and autumn-winter survival in De Leo et al. [10].

De Leo et al. [10] ran their model with and without hunting at different hunting rates. Similarly, we ran the same model using their observed hunting-rate estimate (14.8%) and then removed the effect of lead shot and poisoning from adult mortality, and adjusted fecundity for chick mortality. For adults, we first removed the upper and then the lower bound of mortality caused by lead shot ingestion from the autumn-winter mortality rate in the model, because most lead shot mortality occurs during those seasons. We then additionally removed the mortality from secondary poisoning. We assumed a constant percentage of partridges ingest lead shot or were poisoned by pesticides. Following De Leo et al. [10] and Smart et al. [9], we incorporated joint probabilities of survival to account for the fact that, if all lead shot or poison is removed from the environment, some birds that would not now die of lead shot would still die of other causes. We used the following joint probability equation from a red kite study by Smart et al. [9] to calculate survival (S) in the absence of lead shot ingestion and secondary poisoning:

S=1−[(M⋅n)(M⋅i)+M⋅n] (1)

where M = observed overall mortality rate, i = proportion of total mortality caused by poisoning, and n = proportion of total mortality attributable to other causes. Because n and i are not necessarily constant in density-dependent models but proportion of birds dying due to poisoning (M⋅i) is assumed to be constant, the M used in the M⋅i term was calculated for the relative density at which i was measured. Density was assumed to be at a steady-state population size in the 1960s when i was reported at the 4% level we modeled (i.e., the period when i was highest in southeastern England [20]). Notably, this adjustment changed the outcome very little, and i could have been considered constant.

For the partridge model, we ran Monte Carlo simulations with and without poisoning in @RISK (v. 6.1.2) using 10,000 iterations per simulation to generate (1) mean and 95% confidence intervals for population sizes each year up to 50 years and (2) probabilities of quasi-extinction. The quasi-extinction threshold was set to 5 individuals/km^2^ (mid-range of De Leo et al. [10] quasi-extinction thresholds), which represents approximately 150 individuals on large hunting estates of 3,000 ha. Initial population density was set at 42.8 partridges/km^2^, which was the level observed in some European areas in early spring just before the modeled period (e.g., in the UK [10]). Of a sample of 30 baseline trajectories run, we selected one that is most similar to the observed declining trend during this period (observed data in [10]), and then removed the lead shot and poisoning effects from that one trend as well as from the mean trend to evaluate changes.

To model changes in chick survival from ingestion of lead shot, we first identified the maximum spring adult survival and maximum fecundity rates that produce the De Leo et al. [10] model’s maximum population growth factor in spring of 3.23. The maximum spring survival selected (93.3%) was the mean maximum male and female spring survival in the Potts [24] grey partridge population model at very low bird densities with little competition. The maximum fecundity selected was 2.29 female chicks/adult female, the estimate required to obtain the De Leo et al. [10] maximum spring growth factor using Potts’ [24] survival estimates assigned to all 5 adult age classes. Then, using Eq 1, we removed mortality caused by lead shot ingestion from baseline chick mortality estimated to be 64% from Potts’ [24] model. Removal of lead shot mortality increased the maximum and density-dependent spring growth rates in the standard De Leo et al. [10] model. For the sensitivity analysis, we increased fecundity by an additional 5 and 10%.

#### Common buzzard and red kite models

For the raptor models, we initially constructed each model in RAMAS [39], incorporating density dependence and stochasticity, similar to the partridge model. We used life history and population data from study areas expected to have minimal exposure to lead shot ingestion. For the buzzard, the data came mainly from study areas in Germany (supplemented with data from Finland) that are expected to have low lead shot ingestion because of much lower lead shot usage compared to the UK and Spain [J. Streitberger, AFEMS, Brussels, Belgium, personal communication, 2010]. For the red kite, we used a study area in Wales that was far from hunting estates [17, 21]. We assumed populations in these areas represented the scenario of no ingestion of lead shot, though some minimal amount of lead shot ingestion may have occurred. Therefore, in contrast to the approach of subtracting mortality for the partridge, lead shot mortality was added to the baseline model representing observed conditions for the raptors. We compared population trajectories with (lead shot added) and without (baseline) lead shot ingestion. Birds were exposed to poisoned bait during the baseline modeled period [40]. Therefore, in a separate analysis, we subtracted mortality caused by poison from the baseline model, and we compared population trajectories with (baseline) and without (poison subtracted) poison bait mortality. The trajectory without lead shot or poison ingestion can be compared to the trajectory with both to evaluate the two stressors combined. The parameters for the model were obtained from the literature for the buzzard and kite, as described below.

For the buzzard model, density-dependence was incorporated using the theta-logistic equation [41], which predicts population size (N_t+1_) each subsequent year (t+1) as follows:

$$N_{t+1}=\lambda_{\text{max}}\text{exp}\left[-\text{ln}(\lambda_{\text{max}}){\left(\frac{N_{t}}{K}\right)}^{\theta }\right]N_{t}$$ (2)

where N_t_ is the population size at time t, λ_max_ is the maximum geometric population growth rate factor, K is the carrying capacity, and theta (θ) is the shape of the declining density-dependent curve (ln[λ_t_] vs. N_t_).

**Fig 2:**
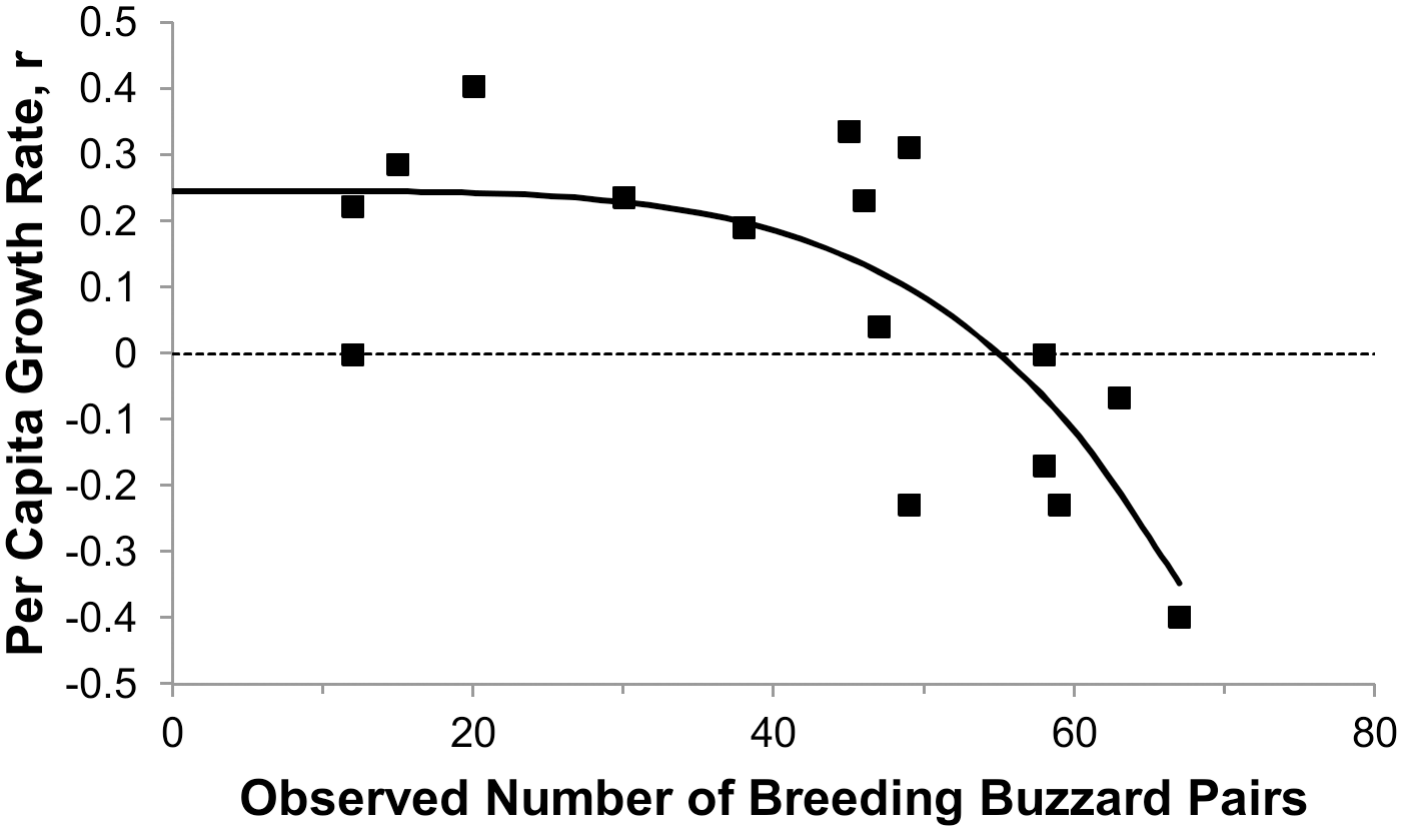
Relationship between per capita growth rate, r, and number of common buzzard breeding pairs in Finland study area [42]. r = lnλ, where λ is the annual population growth factor. This relationship is represented by the theta-logistic equation re-written to calculate the population growth rate as r = r_max_(1 –(N/K)^θ^), where N = population size, K = carrying capacity of 1750, r_max_ = r at N = 0. The θ of the curve is 4.45. This high θ indicates r does not decrease much until density is close to K, the steady-state population size (i.e., where the curve crosses the r = 0 line).

For the buzzard, the λ_max_ that was entered into the theta-logistic equation was the average λ_max_ of three observed time series for breeding common buzzards at locations or during time periods when lead shot densities were low (λ_max_ = 1.28 [42], 1.26 [43], and 1.17 [44], average = 1.2). The first two estimates were calculated using methods of Sibly et al. [41] for complete time series of populations growing from low population sizes to carrying capacity, whereas the third estimate was calculated using the exponential equation applicable for an expanding time series with large data gaps. Using methods in Sibly et al. [41], we fit the most complete time series in Reif et al. [42] to a theta-logistic model to obtain a θ of 4.45 (Fig 2). Because a nonlinear theta-logistic response can not be easily modeled in RAMAS, and beta functions (which model survival well) were unavailable in RAMAS but available in PopTools [45], we replicated the RAMAS model structure and output and ran the final models in PopTools.

We selected the mean density of the buzzard breeding population in an 11-year study conducted in Germany [46, 47] as both the initial population density and the deterministic carrying capacity density of breeding birds (35/100 km^2^) because this population was approximately at an equilibrium carrying capacity (i.e., λ = 1). We converted this density to total breeding pairs in a population covering 10,000 km^2^, resulting in an initial population size (N_0_) of 1,750 breeding pairs (i.e., N_0_ = K = 1,750 pairs). The 10,000 km^2^ area encompasses the dispersal distance of the majority of juvenile birds (most disperse < 100 km [44]) and is large enough to have the population remain extant through time [48]. Thus, it should adequately represent sustainable populations in conditions with minimal or no lead shot ingestion. Note that the modeled mean value of the stochastic equilibrium size over many years at baseline conditions will be lower than the deterministic steady state estimate (< 1,750 pairs) because, during fluctuating stochastic simulations, density dependence depresses population growth more strongly at population densities that are above carrying capacity than it stimulates population growth at population densities below carrying capacity.

Mean survival and fertility were entered into a one-sex, pre-breeding Leslie transition matrix [49]. The matrix was multiplied by a vector of age-class-specific population sizes, as follows:

$${\left[\begin{array}{c} N_{1}\\ N_{2}\\ N_{3}\\ N_{4}\\ N_{5}\\ N_{6+} \end{array}\right]}_{t+1}=\left[\begin{array}{cccccc} F_{1} & F_{2} & F_{3} & F_{4} & F_{5} & F_{6+}\\ S_{1} & 0 & 0 & 0 & 0 & 0\\ 0 & S_{2} & 0 & 0 & 0 & 0\\ 0 & 0 & S_{3} & 0 & 0 & 0\\ 0 & 0 & 0 & S_{4} & 0 & 0\\ 0 & 0 & 0 & 0 & S_{5} & S_{6+} \end{array}\right]\cdot {\left[\begin{array}{c} N_{1}\\ N_{2}\\ N_{3}\\ N_{4}\\ N_{5}\\ N_{6+} \end{array}\right]}_{t}$$

where N_x,t_ is number of x-year-old females at time t (i.e., N_1,t_ is number of 1-year-old females at time t). Survival probabilities of a female of age x between time t and t+1 are denoted as S_x_, and fertility (females at time t+1 that were produced by females at time t) as F_x_. For the oldest year class, we combined all adults age 6 and older, which technically converts the Leslie matrix to a 6 x 6 Lefkovitch matrix [49]. The vital rates in the matrix (F_x_, S_x_) were developed for a relatively stable population at carrying capacity from the German data in Krüger and Lindström [46], supplemented by the Kenward et al. [50] radio-telemetry data for proportion breeding and relative survival of the early age classes of a stable population (Table 1). The survival and fertility estimates in the matrix from these studies were adjusted slightly to produce λ = 1 at carrying capacity. Applying the theta-logistic equation parameters, each age-specific survival and fertility rate was then modeled as density-dependent, which is supported by other raptor studies [51–53]. The modeled relationships of these vital rates versus density produce the relationship observed between r and population size (S2 Fig).

**Table 1:** Mean vital rates (standard deviations in parentheses) used in matrices for the common buzzard and red kite.

Age	Common Buzzard^a^		Red Kite^b^	
x	F_x_	S_x_	F_x_	S_x_
**1**	0.00 (0.00)	0.75 (0.10)	0.00 (0.00)	0.79 (0.08)
**2**	0.03 (0.03)	0.88 (0.10)	0.07 (0.09)	0.79 (0.08)
**3**	0.06 (0.07)	0.88 (0.10)	0.41 (0.09)	0.86 (0.08)
**4**	0.15 (0.26)	0.88 (0.10)	0.41 (0.09)	0.86 (0.08)
**5**	0.35 (0.29)	0.88 (0.10)	0.41 (0.09)	0.86 (0.08)
**6+**	0.44 (0.29)	0.72 (0.10)^c^	0.41 (0.09)	0.79 (0.08)^c^

^a^Initial rates for steady-state population size (produces λ = 1).

^b^Initial rates for low-density population size (produces λ = 1.065).

^c^Some studies assume survival constant for adults [8, 50], but long-term studies [46] show survival decreases at older age classes [6+].

**Fig 3:**
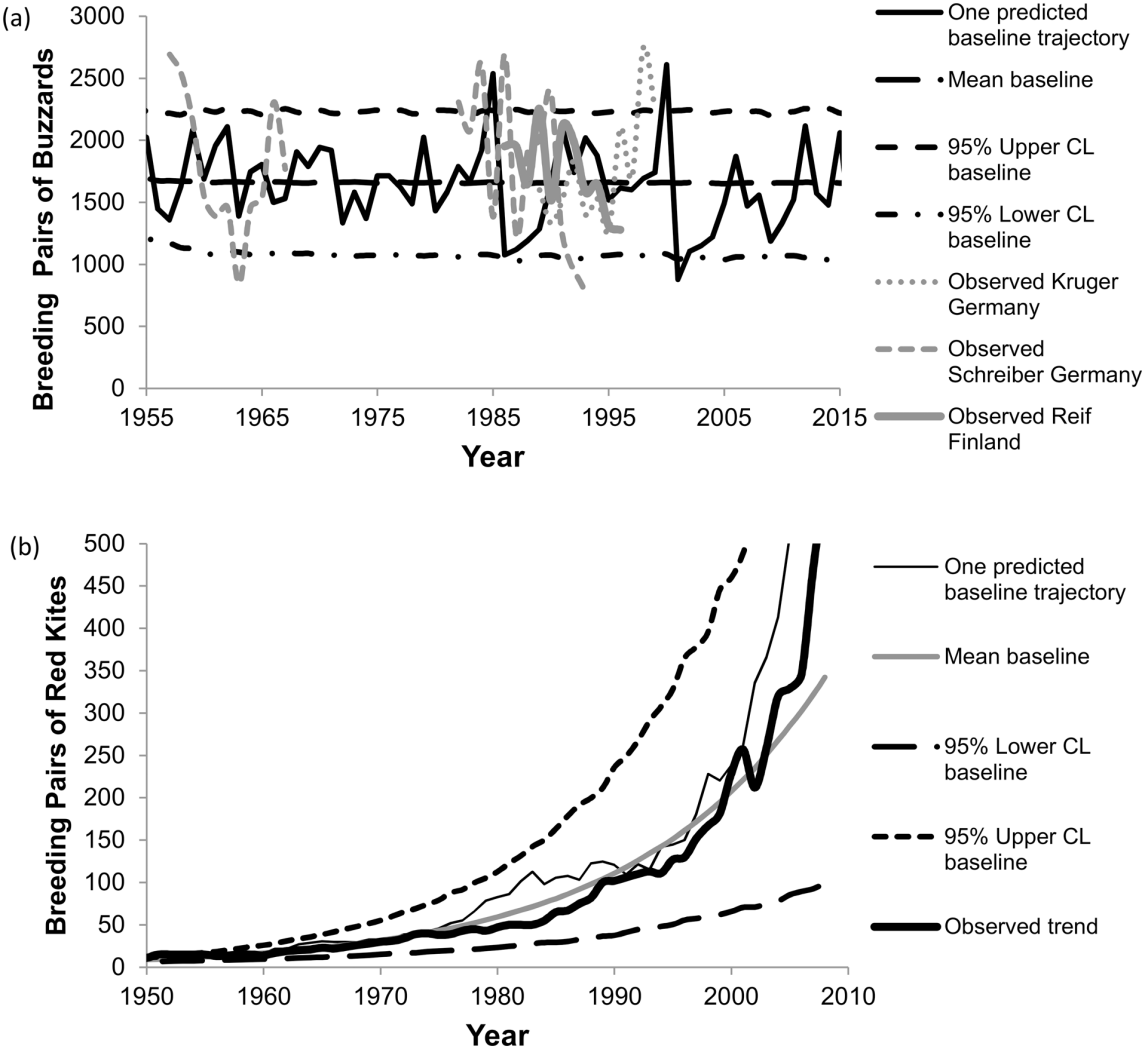
Comparison of the baseline-model population trend (mean and its 95% confidence limits) to the observed population trajectories of (a) common buzzards and (b) red kites. One typical stochastic-predicted trajectory is also included to show the variation being modeled. The three observed trajectories are from Reif et al. [42], Schreiber et al. [43], and Krüger and Lindström [46].

To model environmental variation, the vital rates in Monte Carlo simulations were randomly selected each year from lognormal distributions of fertility rates (truncated to maximum rates possible) and from beta distributions of survival rates. We used temporal standard deviations of fecundity derived from Krüger and Lindström [46] to model environmental stochasticity (Table 1). Temporal standard deviations of survival for buzzards were unavailable and were set similar to those in the red kite model, adjusted upward slightly to account for high variability observed in the buzzard population size. The variability of the population size around carrying capacity in one typical baseline trajectory of the buzzard model over the modeled period is large, but was similar to three observed, published buzzard trajectories in Germany and Finland during the period of 1955 to 2015 (Fig 3a). We recognize that some of the variability is probably due to sampling error, but our intention was to superimpose effects of ingestion of lead shot and poison on top of observed baseline trajectories that include such error. These trends probably also include fluctuations in immigration and emigration, but overall standard deviations in observed and modeled trends were large because the population size of buzzard breeding pairs fluctuates with availability of their food sources (e.g., fluctuating vole populations [18]).

The model structure for the red kite was the same as the buzzard model but re-parameterized for the kite. Long-term demographic information for the kite is available from 1946 to 2008 in Wales, and we modeled that entire period. These years represent a period of exponential growth following implementation of protective measures for the birds [17, 44]. Survival and reproduction did not decrease as population size increased in the Welsh population (data in [17, 54]), supporting that the growth during the modeled period was density-independent. The population growth rate (λ) during the period up to 1993 was 1.05 [17]. Since 1993, the growth rate has increased as the kite population has expanded into more productive, low-elevation areas [40]. Using published monitoring data from 1946 to 2000, the growth rate was 1.065 [17, 40]; and additional online data from the Welsh Kite Trust [54] indicates it grew even faster between 2000 and 2008, but some of the recent increase might also be from stochastic variation. We parameterized the baseline model to grow deterministically at a rate of 1.065, which best fits the majority of the observed growth trajectory from 1946 to 2008. This growth rate is produced by fertility and survival rates (Table 1) estimated after examining data in Newton et al. [17], supplemented by breeding proportions estimated in Tenan et al. [8]. The data were entered into a pre-breeding, one-sex 6 x 6 matrix with standard deviations to model stochasticity in PopTools.

The entire UK population of red kites had declined to only 14 breeding kites in Wales in 1946 [17]. The initial breeding population size (N_0_) in the model was set to this level of 7 breeding pairs for a 10,000 km^2^ study area in 1946, the starting year of the population trajectory. The output of the baseline model was similar to the observed population trajectory for this study area (Fig 3b).

To avoid the modeled Welsh population increasing to unrealistic levels by 2008 after removing mortality caused by bait poisoning from annual total mortality, density dependence was incorporated into the model (i.e., we incorporated λ_max_ = 1.065, θ = 5.58, and K = 1,750 into a theta-logistic equation). Red kites have been increasing in the Welsh core area and are slowly expanding outward, but growth is expected to slow when the population approaches high densities at steady state. The carrying capacity of the red kite breeding population in the area monitored in Wales is unknown. For this modeling exercise, we set it at the same level as the buzzards (i.e., 3,500 breeding birds, assumed to be 1,750 pairs), which is approximately 1/6 of the projected carrying capacity of 20,000 breeding red kites for all of the UK [55]. We set λ_max_ equal to the observed growth rate of 1.065, which represents growth at low densities with low intraspecific competition. To estimate θ, population trajectories that increase from low densities to carrying capacity are needed but are not available for the red kite in the literature. However, the snail kite (*Rostrhamus sociabilis*) in Florida has an estimated θ of 5.58 (we derived this value by applying the Sibly et al. [41] method to data in [56]; S3 Fig), indicating kite populations may not experience density-dependent growth until they increase to near steady state, similar to the buzzard. Therefore, a high θ of 5.58 was applied in the theta-logistic regression in the red kite population model. This θ produces no density-dependence at the low, currently-observed kite densities.

The raptor models were run with and without ingestion of lead shot and poison during the same period as the observed trajectories, using 10,000 Monte Carlo iterations per simulation. For the scenario with lead shot ingestion, we removed the proportion of the raptor population estimated to die from lead shot ingestion (assumed to be constant) by applying the upper and lower bounds of that estimate. We calculated the total annual survival in the presence of lead shot ingestion using the following equation [57]:

S=1−{M⋅p+M⋅i−[(M⋅p)(M⋅i)]} (3)

This equation contains the same terms defined in Eq 1, except p is the fraction of mortality attributed to other causes in the absence of lead shot ingestion. For the scenario of removing bait poisons that affect the Welsh population, we used Eq 1. We calculated M⋅i in both equations at observed relative densities when i was measured (low density for the kite and at steady state for the buzzard; but the model was relatively insensitive to the density selected). For the fecundity sensitivity analysis, we decreased fecundity in the raptor models by 5%.

The quasi-extinction threshold for the common buzzard was set at 7 breeding birds/100 km^2^, equivalent to 350 breeding buzzard pairs in the modeled 10,000-km^2^ study area and equal to one-fifth of the current equilibrium population size. Because the current red kite population is already below such quasi-extinction levels, we used a quasi-extinction threshold for the red kite of 6 breeding pairs in the 10,000 km^2^ study area (i.e., only slightly less than the minimum of 7 pairs observed in 1946 in that population).

## Results

### Grey partridge

**Fig 4:**
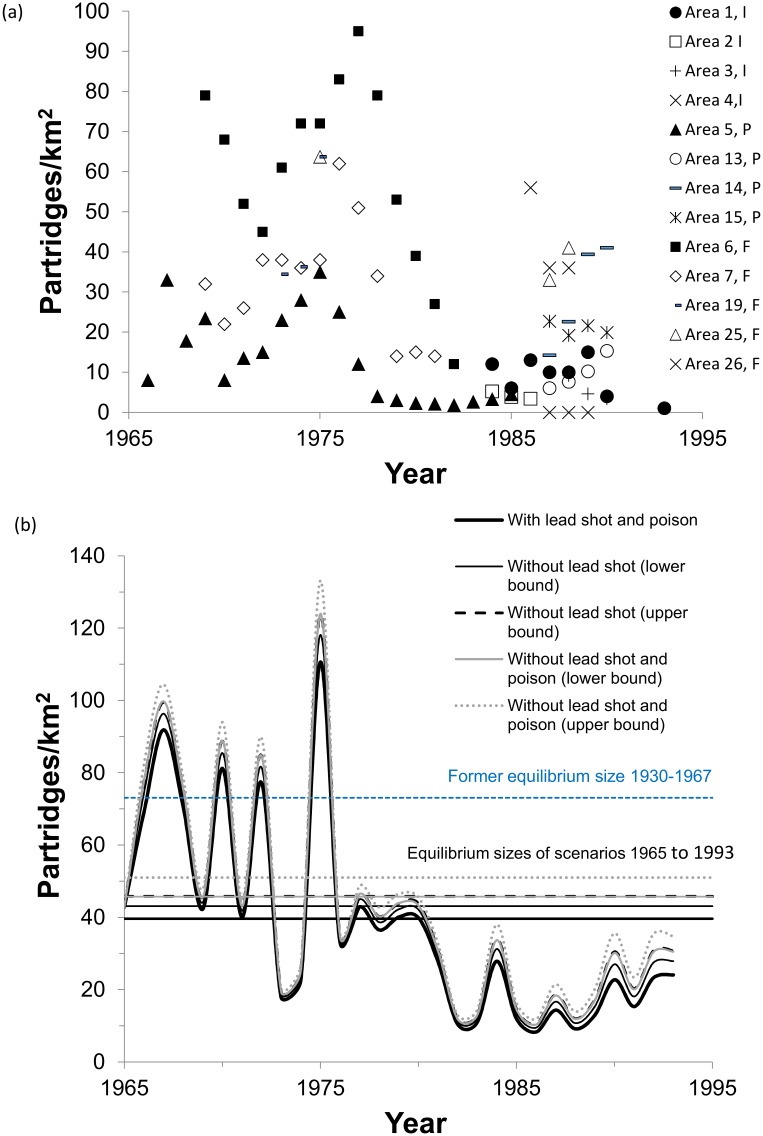
Grey partridge observed and modeled population trends. (a) Observed declining grey partridge population trends shown in different areas of continental Europe (I = Italy, P = Poland, F = France [10]). (b) One modeled declining partridge trajectory shown for four scenarios of the population in continental Europe fluctuating around the steady-state equilibrium population size (horizontal line) with and without ingestion of lead shot and pesticide poisons. Lower and upper bounds represent removal of ingestion of lead shot causing 4 and 7% of the mortality, respectively. Shown for comparison is the estimated higher equilibrium size (in blue) during the early 20^th^ century (based on UK data [10]).

The De Leo et al. [10] partridge model predicts an equilibrium population size that represents the maximum number of partridges the environment can hold on average, given the various stressors present. The model output for one trajectory that is similar to observed trends shows that the baseline population exposed to lead shot and poison fluctuates greatly around that steady-state equilibrium level (observed trends in Fig 4a, model output in Fig 4b). The baseline model output with both lead shot and poison present indicates a 16% probability that stochastic fluctuations will drive the population to near extinction (see quasi-extinction rates in Table 2). The population has been declining since the early 20^th^ century. The steady-state population size in continental Europe was probably much higher in the early 20^th^ century than in the latter part of the century, possibly at similar levels to the estimated equilibrium population density in the UK in the early 20^th^ century (73 birds/km^2^ with observed harvest rates, Fig 4b, based on UK model in [10]). However, no early 20^th^ century datasets for continental Europe are available to verify this.

**Table 2:** Model outputs for baseline^a^, lead shot, and poison-ingestion mortality scenarios.

Output Parameter	With lead shot & poison	With poison but no lead shot	Without poison & lead shot
**Grey Partridge (Continental Europe)**			
Probability of quasi-extinction to < 5 birds/km^2^ in 50 years	0.16	0.085^b^ (0.0*7–0*.*10*)	0.045^b^ (0.0*3–0*.*06*)
Mean steady-state population size (birds/km^2^)	40	44.5^b^ (*43–46*)	48.5^b^ (*46–51*)
	(CL^c^ = 10–107)	(CL = 10–113)	(CL = 11–123)
**Common Buzzard (Germany)**			
Probability of quasi-extinction to < 350 breeding pairs/10,000 km^2^ in 50 years	0.01	0.01	0.01
Mean steady-state breeding pairs/ 10,000 km^2^	1650^b^ (*1643–1656*)	1656	1666
	(CL = 1068–2202)	(CL = 1039–2227)	(CL = 1015–2245)
**Red Kite (Wales)**			
After reached > 50 pairs, probability of quasi-extinction to < 6 breeding pairs/ 10,000 km^2^ in 50 years	<0.0001	<0.0001	<0.0001
Deterministic maximum population growth rate (lower to upper bound)	1.041^b^ (*1*.*035–1*.*046*)	1.065	1.118

^a^ Baseline (observed conditions) for partridge is with lead shot and poison (second column of table), and for raptors is without lead shot but with poison (third column of table).

^b^ Midpoint of lower- and upper-bound estimates presented with lowest and highest bounds italicized in parentheses if they differ (bounds are only needed for the predictions, not for the observed baseline condition).

^c^CL = 95% confidence limits.

Removal of lead shot and poison ingestion from this baseline scenario shows the effect of such poisoning (Fig 4b). Lead shot ingestion alone reduced deterministic adult survival in the autumn-winter period at steady state by at most 2% (e.g., from 0.494 down to 0.486 for upper-bound lead shot effects). Using midpoints of upper- and lower-bound estimates in Table 2, the modeled lead shot ingestion reduced the steady-state population size during the period 1965 to 1993 by 10% from 44.5 (with only poison present) to 40 birds/km^2^ (with lead shot and poison present; Table 2). Probability of quasi-extinction in 50 years, starting in 1965, increased from 8.5 to 16% when the effect of lead shot ingestion was added to the effect of poison (Table 2). The combination of lead shot ingestion and secondary poisoning from pesticides reduced the steady-state population size by 18% from 48.5 (without either stressor) to 40 birds/km^2^ (with both stressors) and increased the probability of quasi-extinction to < 5 birds/km^2^ from 4.5% (without either chemical stressor) to 16% (with both chemical stressors; Table 2). The observed trajectories support this result, showing some populations in continental Europe declined below the quasi-extinction threshold of 5 individuals/km^2^ (Fig 4a). In the presence of both stressors, autumn-winter survival decreased by 4% (upper bound) to 7% (lower bound).

### Common buzzard

**Fig 5:**
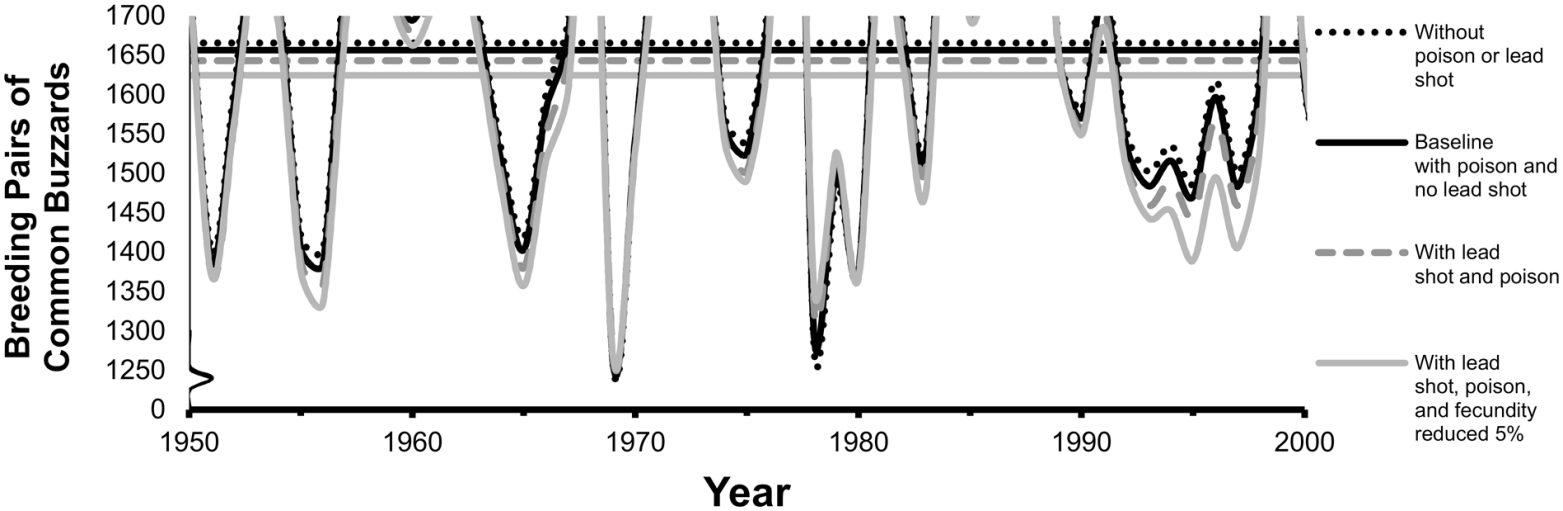
Comparison of the common buzzard steady-state population size and one example trajectory for four scenarios. The steady state (horizontal line) is the mean of 10,000 trajectories. Scenarios include with and without ingestion of lead shot (shown at upper-bound rate of 5% of total mortality) and bait poison (at 4% of total mortality), plus the sensitivity analysis with 5% reduction in fecundity added to mortality effects. The upper part of the example trajectory is not shown to amplify the lower part of the trajectory.

At upper-bound estimates of exposure, annual survival decreased by 0.6% (higher-survival age classes) to 1.5% (lower-survival age classes) with lead shot exposure alone and by almost the same amounts (0.5–1.1%) with poison alone. The midpoint of the modeled equilibrium size of breeding pairs of common buzzards shifted downward very little (by no more than 1%, Fig 5) when lead shot and poisons were included in the model (Table 2; only upper-bound effects shown in Fig 5 because the lower bound resulted in no effect to buzzards). Probability of quasi-extinction to < 350 pairs/10,000 km^2^ remained low at 1% in all scenarios (Table 2).

### Red kite

**Fig 6:**
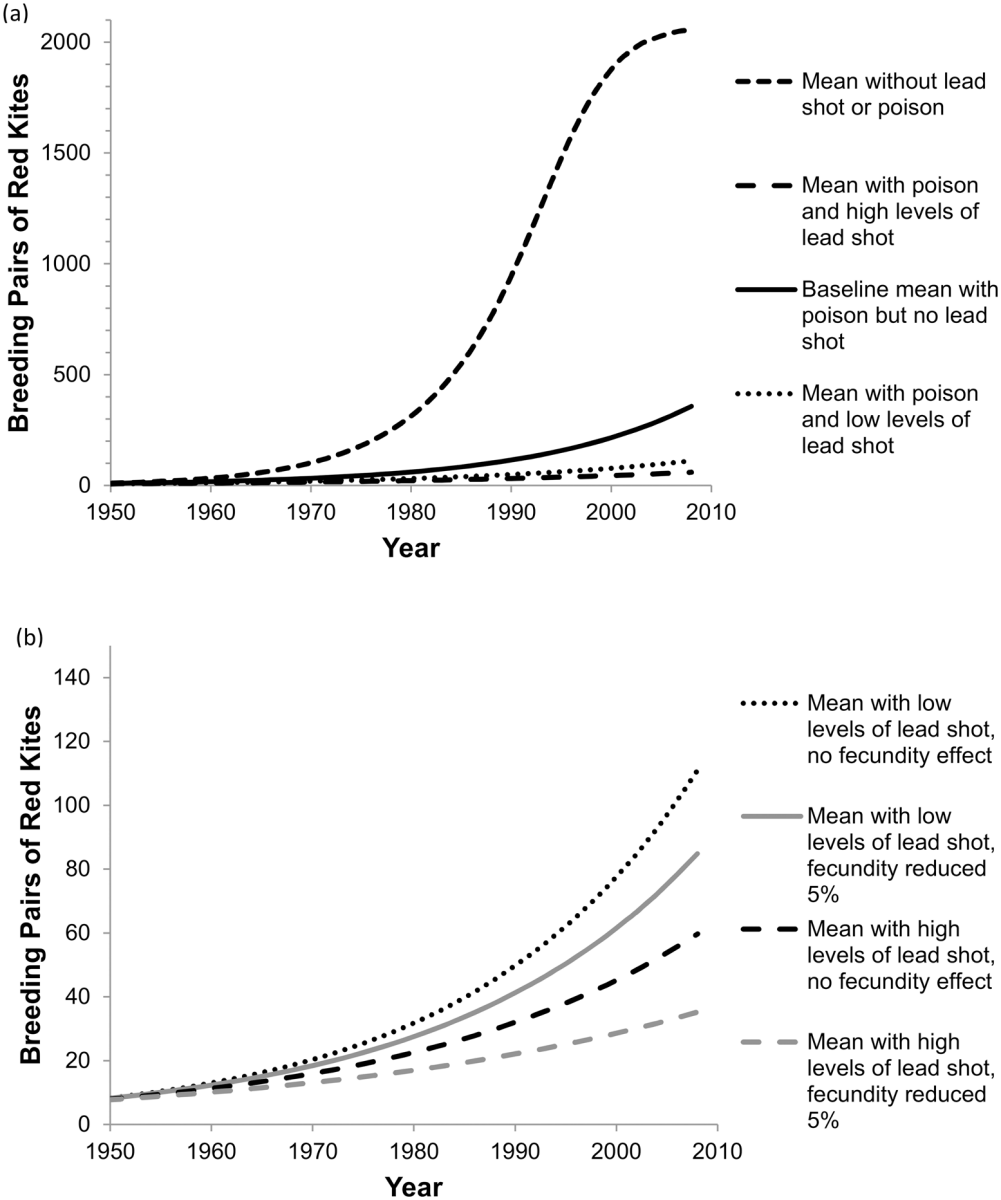
Comparison of red kite mean population trajectories (of 10,000 runs) for various scenarios. (a) Four scenarios shown with or without lead shot ingestion or poison. (b) Sensitivity analysis shown comparing 0 and 5% reduction in fecundity. All scenarios in (b) still include lead shot and poisons affecting mortality but different fecundity effects. Low and high levels of lead shot refers to scenarios of 9% (lower bound) and 16% (upper bound) of total mortality attributable to lead shot ingestion added to the baseline condition.

The Welsh red kite population increased during the modeled period and probably currently is still far from reaching a steady-state carrying capacity. When lead shot ingestion was added, the modeled population growth rate decreased from 1.065 to a midpoint estimate of 1.041 (range 1.035–1.046, Table 2). Lead shot reduced annual survival of age classes by a small amount, between 1.4% and 3.9%. With lead shot ingestion added, the population would still grow and have < 0.01% probability of quasi-extinction to < 6 breeding pairs (Table 2), but would grow slower (Fig 6a). If lead shot and poison ingestion were removed, the growth rate would increase greatly to 1.118 (Table 2), with the population almost reaching a steady state by 2008 (Fig 6a). Bait poisons alone reduced annual survival by 6.4 to 9.6%.

### Sensitivity Analysis of Fecundity Effects

**Fig 7:**
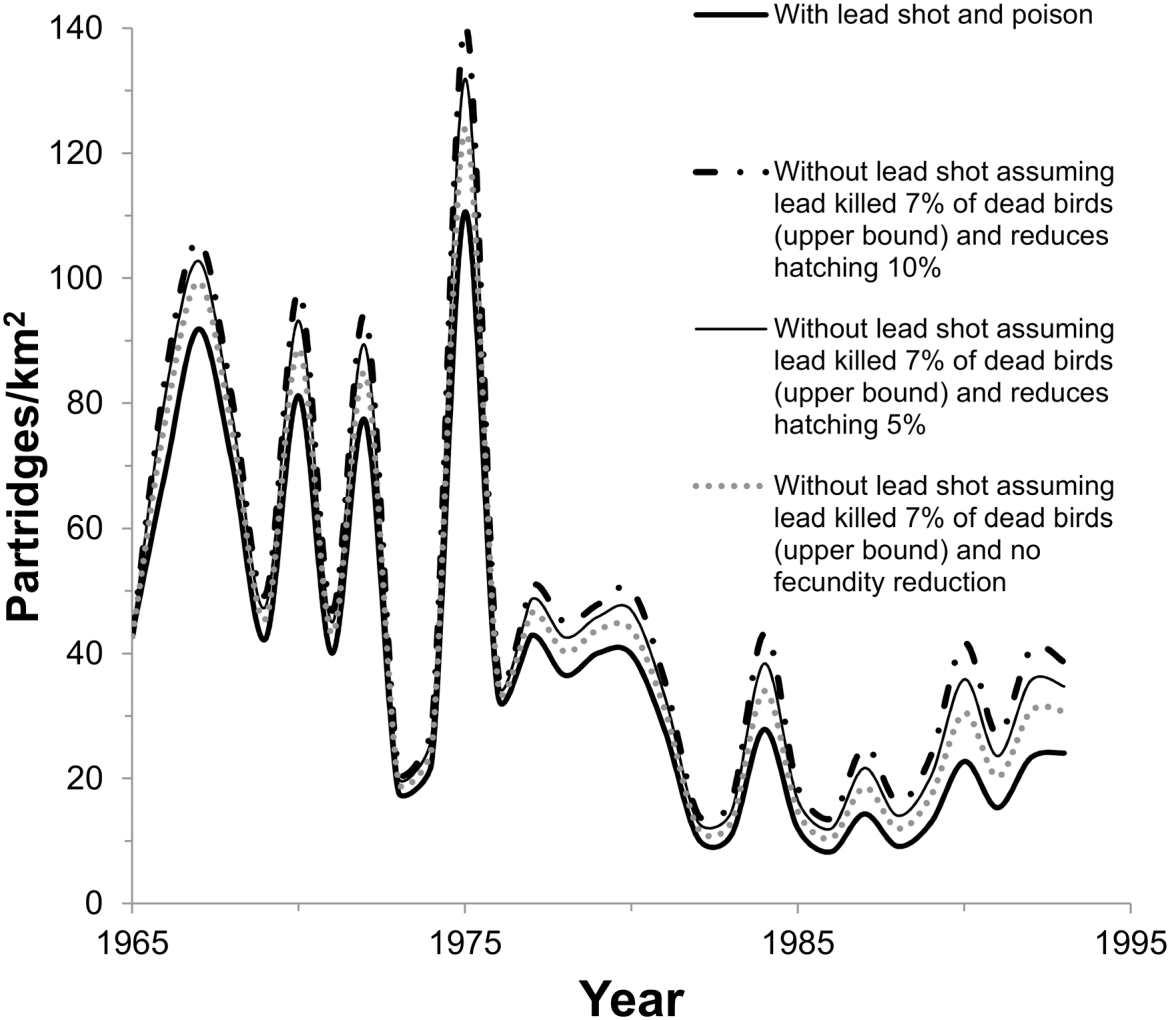
Results of sensitivity analysis, assuming lead shot ingestion additionally reduces fecundity for partridge scenarios in Fig 4b by 5 or 10%. Only the upper-bound estimate is compared to baseline, representing removal of ingestion of lead shot that caused 7% of the mortality.

Although fecundity is presumably reduced when partridge chicks ingest lead shot, it is unknown whether adult ingestion of lead shot or pesticides affects mean clutch size or hatching success in partridge populations (i.e., whether lead shot causes sublethal effects on reproduction). If these substances are ingested at sublethal doses in spring and assumed to additionally reduce partridge reproductive success beyond lead shot effects on chick survival by 5 to 10%, the steady-state population size, with poison mortality still included, would decrease by 16 to 21%, respectively (from 47.5 to 40 or 50.5 to 40 birds/km^2^ using midpoints in Table 3; also see upper-bound effects in Fig 7) and by 6 to 12% from the additional fecundity effects alone (from 47.5 to 44.5 or 50.5 to 44.5 birds/km^2^, compare Tables 2 and 3). Probability of quasi-extinction to < 5 birds/km^2^ could increase from a low range of 2.5 or 5% up to 16% (midpoints in Table 3).

**Table 3:** Results of sensitivity analyses in which the non-baseline^a^ scenario has fecundity further adjusted for impacts (by lead shot or poison) by 5 or 10% for the grey partridge and by 5% for raptors.

Output Parameter	Fecundity adjustment	With lead shot & poison mortality	With poison mortality but no lead shot
**Grey Partridge**			
Probability of quasi-extinction to < 5 birds/km^2^ in 50 years	5%	0.16	0.05^b^ (0.0*4–0*.*06*)
	10%	0.16	0.025^b^ (0.02–0.03)
Mean steady-state population size (birds/km^2^)	5%	40	47.5^b^ (*46–49*)
	10%	40	50.5^b^ (49–52)
**Common Buzzard**			
Probability of quasi-extinction to < 350 breeding pairs/10,000 km^2^ in 50 years	5%	0.01	0.01
Mean steady-state breeding pairs/10,000 km^2^ (95% confidence limits)	5%	1640^b^ (*1624–1656*)	1656
**Red Kite**			
After reached > 50 pairs, probability of quasi-extinction to < 6 breeding pairs/10,000 km^2^ in 50 years	5%	<0.0001	<0.0001
Deterministic maximum population growth rate	5%	1.034^b^ (*1*.*026–1*.*041*)	1.065

^a^Baseline (observed conditions) for partridge is third column in table and for raptors is fourth column. The fecundity adjustment does not apply to a species’ baseline (observed) scenario.

^b^Midpoint of lower- and upper-bound estimates is presented with lowest and highest bounds italicized in parentheses.

In contrast, when common buzzard fecundity was assumed to be reduced 5% in addition to the reduction in survival from poison at baseline, the equilibrium breeding-pair size decreased by <1%, from 1,656 at baseline to 1,640 breeding pairs (midpoints in Table 3). With lead shot mortality added to the poisons and reduction in fecundity, the equilibrium size decreased to 1,624 breeding pairs (Fig 7). Probability of quasi-extinction was 1%, the same as the baseline scenario.

For the growing red kite population, adding a 5% reduction in fecundity decreased the population growth rate from 1.065 at baseline to 1.034 (Table 3, Fig 6b). The fecundity reduction alone decreased the population growth rate from 1.041 to 1.034 (compare Tables 2 and 3). However, the red kite population was still increasing and probability of quasi-extinction was low (<0.01%) after reaching 50 pairs.

## Discussion

Widespread practices that expose bird populations to chemical stressors in Europe, such as use of lead shot, pesticides, and poisoned baits have the potential to alter population dynamics of susceptible species, particularly because these substances are increasing or continue to be released in large amounts in the environment. Mean lead shot densities in soil on small-game hunting areas in Europe have been reported to range from approximately 5,000 to 75,000 lead shot pellets/ha [4]. Because up to 80% of the land in the EU could be used for small-game hunting and because lead shot accumulates in the soil through time, the potential for avian exposure to lead shot in the EU environment could increase over time. In addition to the lead exposure, coating of seeds with some potentially toxic pesticides continues in Europe, exposing granivorous birds [31]. Furthermore, mortality of raptors feeding on poisoned baits is often reported as high in some European countries, restricting species ranges or the population growth rate [8, 28–29, 58]. We evaluated the effects of lead shot ingestion alone and in combination with these poisons on three terrestrial bird species to demonstrate a modeling method that can assist in understanding the implications of multiple chemical stressors on population size, growth, and extinction rates. Though the direction of trends did not change, our modeling results illustrate that effects of lead shot and other poisons were larger for the declining and recovering populations of grey partridge and red kite that we modeled than for the large, stable population of the common buzzard.

### Grey partridge

Though population models have much uncertainty, our results suggest the combination of lead shot and other poisons may have accelerated the decline of the grey partridge population in continental Europe. The grey partridge steady-state population size decreased from the early to the latter part of the 20^th^ century, which is primarily attributed to increased pesticide usage that decreased food sources for chicks and to reduced cover for adults [14]. Our model illustrates how lead shot ingestion and more direct effects of poisoning also might have contributed to the decrease. The lead shot ingestion and pesticide poisons combined may have decreased the equilibrium size of the already declining grey partridge population by almost 20% (not including possible hatching effects).

Adding chemicals in the environment to already stressed, declining populations can create greater effects than on healthy populations. For example, De Leo et al. [10] modeled a healthy grey partridge population during the early part of the 20^th^ century in the UK with little exposure to lead shot ingestion [20]. Using the same modeling methods described above for the declining partridge population, we added lead shot ingestion mortality to the non-lead shot mortality already in that UK model using our upper bound of 7% of all mortality assumed to be from lead shot. Adding lead shot ingestion, the probability of quasi-extinction of the healthy population to < 5 birds/km^2^ remained very low (<0.01%). In contrast, it increased from 7 to 16% for the stressed population. Additionally, the upper-bound percentage reduction in steady-state population size was smaller for the healthy population (8% instead of 13% reduction).

Regarding lead shot ingestion alone, the effect was relatively small compared to the high temporal variability observed in partridge populations. France contains more than half of the continental grey partridge population [59], and its variable trends dominate partridge trends in continental Europe. Similar to other European countries [14], French populations of grey partridges may have declined in the late 1900s [59]. Though the trends appeared to have been relatively stable by 1998 to 2012 [60], the populations have very high spatio-temporal variability at the French commune scale (2 to > 100 partridges/km^2^).

**Fig 8:**
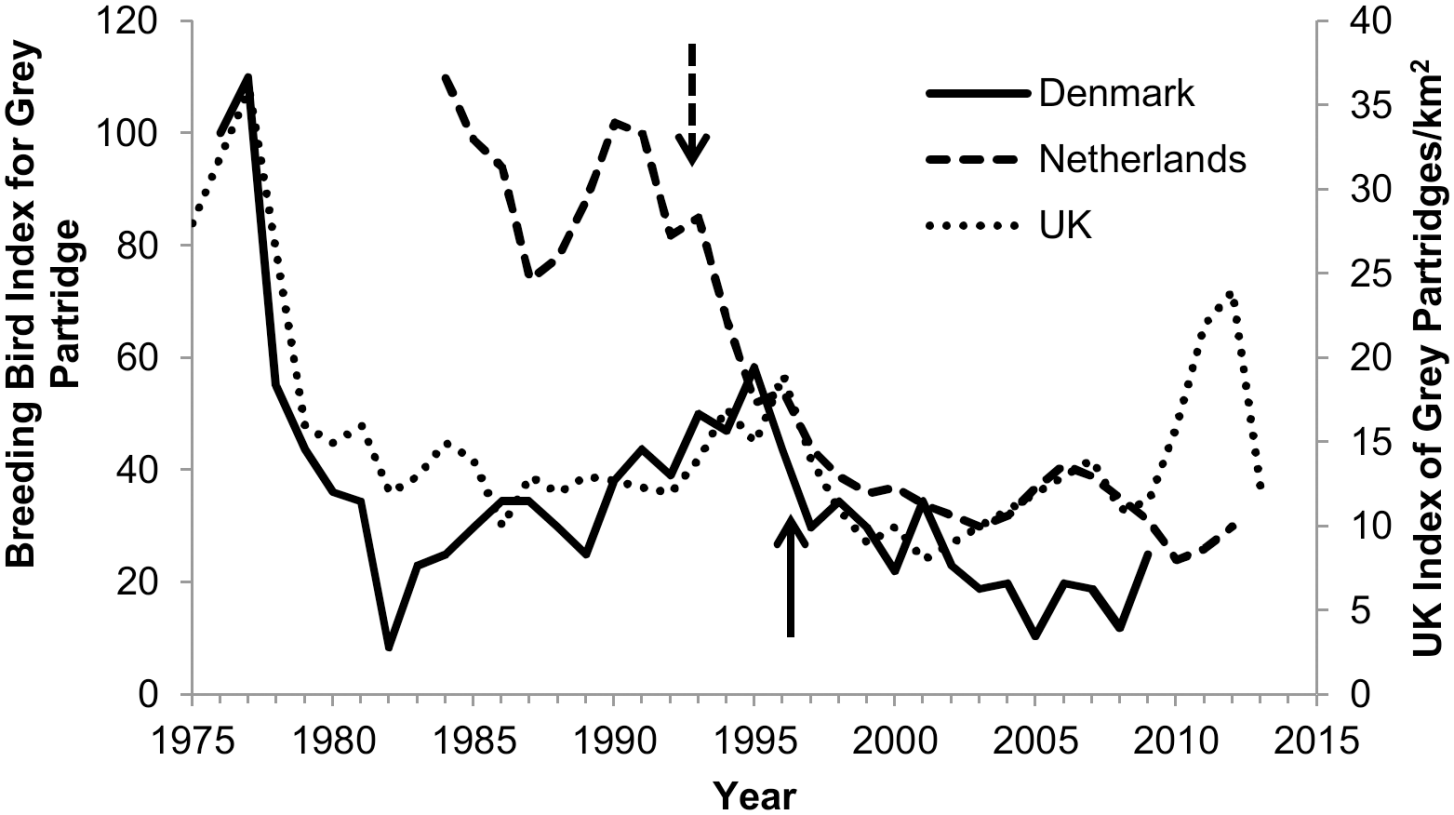
Breeding-bird indices for grey partridge populations in three European countries. The Netherlands and Denmark banned lead shot in 1993 and 1996, respectively (shown by arrows); and the UK did not ban lead shot (UK data are plotted on secondary axis). Data from [61–62, 65].

Such high variability in France and other European countries makes it difficult to identify population changes if lead shot is banned from all hunting, as was the case in the Netherlands and Denmark. These countries banned lead shot in 1993 (Netherlands) and 1996 (Denmark), and the population response is not yet distinguishable (Fig 8, [61, 62]). Lead shot lying on the ground has a 100- to 300-year degradation time [63, 64], but it can become less available to birds if it is buried as new soil accumulates on top or as the lead shot is actively buried (e.g., by cultivation) and not returned to the surface. Therefore, populations could in concept begin recovering after a lead shot ban ceases new inputs of lead into the soil or at least not worsen. If all other factors affecting the population remain relatively constant, the population should improve slowly over long periods of time when compared to trends from other countries that did not ban lead shot. However, during the first approximately 20 years since the total ban on use of lead shot in Denmark and the Netherlands, the partridge populations trends in those countries appear similar to the trend in the UK [65] (Fig 8), where lead shot use has not been banned for hunting in non-wetland areas. After 1995, along with the UK, the Dutch and Danish populations of partridges appear to be fluctuating around an even lower steady state than during the previous 30 years (Fig 8). Considerable uncertainty exists about temporal delays in population response to lead shot bans and masking of population responses by background variability, which is why population models assist in evaluating potential responses.

A population model is a useful tool for assessing relative magnitudes of effects of chemicals such as lead shot ingestion that cause small-percentage effects. Our modeling supplements analyses of population trends that inherently have high variability, which preclude inferences about subtle changes in population density. However, our model assumes the proportion of dead birds diagnosed as dying of a chemical stressor (e.g., lead shot) is known for an area of interest. The proportions of mortality from lead shot ingestion in grey partridges in Europe that have been reported to date in the literature are limited and mostly from England. The estimates of mortality proportions were 3.4% in southeastern England in the 1960s [20]; 2.7% in the same area in England from 1970 to 1992 [20]; 5 to 6% and 3% in Sussex County, England from 1952 to 1977 and from 2003 to 2011, respectively [66]; and approximately 1% in Denmark in the 1970s [67]. Because grey partridges dying from shooting were not included as a cause of death in post-mortems in these literature sources, we added a harvest rate of 15 to 25% (respective rates from late 20^th^ to early 20^th^ century [10]) to estimate these percentages of deaths. Our modeled example of 4 to 7% represents the higher end of the range of these estimates.

Pathologists did not use lead concentrations in bone or liver to corroborate their diagnosis of lead poisoning for grey partridges that died, which creates some uncertainty in the diagnosis and our modeled partridge estimates. Proportion of dead gallinaceous birds in European countries with elevated lead concentrations in bone or tissues could not be found in the literature, though some limited data on prevalence of such concentrations in live gallinaceous birds that were harvested are available. Lead concentrations in bone of live birds shot by hunters were elevated (> 20 mg/kg dw) apparently from lead shot ingestion (based on isotopic signatures) in 3.5%, 4.5%, and 18.4% of red grouse (*Lagopus lagopus scotica*) bones examined from Angus (Scotland), Aberdeenshire (Scotland), and Yorkshire (England), respectively [68]. These live-bird exposure estimates are not useful for modeling gallinaceous birds, however, because they are a snapshot in time and do not represent annual exposure or annual mortality (required in the model). Also, elevated bone concentrations alone are not diagnostic of lead poisoning [68].

Similar to lead shot ingestion, the proportion of grey partridge deaths due to direct pesticide poisoning is also relatively small and difficult to detect, given the variability in population sizes. In Sussex County, 0 to 1% of mortality was from pesticides [66], which is much lower than the estimate of 6% that we used based on necropsies of partridges in France [23]. If we had added to the French post-mortems a 15% hunting mortality (estimate from [10]), the percentage dying from pesticide poisoning would have been slightly lower, approximately 5%. Therefore, our analysis might be illustrating pesticide effects for a high rate of poisoning, and may not represent current exposure in typical partridge populations across Europe. In contrast to direct mortality, indirect effects of pesticides on food sources for partridge chicks have been documented to be large, strongly affecting partridge population trends by reducing chick survival [14, 24].

### Common Buzzard

The common buzzard populations are currently large and stable in Germany, having recovered from lower numbers in the 1950s and 1960s caused by pesticide poisoning [47]. Contrary to the results for the grey partridge, the added lead or poison-related mortality at levels observed in the UK in the 1990s (up to 9% of deaths, Fig 1) resulted in minimal impact on the German buzzard population (≤ 1% decline in population size). Even though it frequently hunts for its food, the common buzzard could be at greater risk of extinction if chemical exposure in carcasses it feeds upon is higher. Illegal poisoning was much higher than our modeled rate in some regions in the past, reported as the cause of death for 54% of dead common buzzards in northeast Scotland from 1964 to 1972 [58]. At such percentages, the German buzzard steady-state population could decrease by one-third based on our model. If poison-related mortality is further increased to 64% of deaths, the German buzzard population could reach quasi-extinction (< 350 breeding pairs/10,000 km^2^) in 50 years.

### Red kite

Based on these modeling results, lead shot and poison slow the recovery of the red kite in the UK. Red kite populations are vulnerable when at low population sizes, but the Welsh population size currently is greater than 600 birds, sustainable and growing. The modeling results support that the population would probably continue to be sustainable even if higher levels of lead shot ingestion (ultimate cause at 16% of mortality) were added as a stressor. However, our models show addition of mortality from lead shot ingestion at such high rates would reduce the already slow growth to a few percent less per year (from 6.5%/yr to 3.4%/yr). Using a diffusion model, Lensick et al. [44] also found minor changes in survival can substantially alter the rate of range expansion of raptors. Though our results are based upon the population dynamics of wild UK red kite populations, they may also apply to introduced populations. Establishment of red kite populations introduced in various regions in the UK since 1989, though considered successful [40], also may have been slowed by ingestion of lead shot [69]. We also caution that our modeling results showing continuing growth are based on growing populations and do not apply to other red kite populations in Europe that are declining or at low numbers [70].

Mortality of red kites from lead shot ingestion probably varies greatly by region. The upper bound percentage modeled for lead shot ingestion as cause of death was 16% based on the percentage of 44 dead kites with liver concentrations greater than the subclinical liver threshold, which is very close to the 18% of bones of 86 dead red kites from the same English study area that were above the bone threshold concentration of concern (20 mg/kg; [21]). These data support that this region has high lead shot exposure. Other European study areas reported that percentage of mortality of raptors from lead shot ingestion is much lower than the 9% lower bound proximal rate used in our model. Pathologists reported these other rates were ≤ 1.6% for red kites in France [71] and ≤ 2.4% for raptors in Spain [72, 73]. Possibly, raptors in other areas may be less affected by lead shot ingestion than our model output indicates. Mortality of the Welsh red kites from lead shot ingestion would have to be 31% of total mortality in 1950 (i.e., in addition to poison-related mortality) in order to cause the population to decline in our deterministic version of the model, and that mortality rate is many times higher than currently-reported levels.

Illegal and secondary poisoning from baits has a much larger impact on red kite populations than lead shot ingestion. Such poisoning may have already slowed annual growth of the Welsh red kite population from 11.2% to 6.5%, if 46% of deaths in the modeled period (based on rates in England [21]) had been from poisoning. Recent poisoning percentages for the Welsh population are reported to be similarly high, over half of all mortalities [74]. If these kites had not been exposed to illegal or secondary poisoning in baits, our model indicates the population size could have recovered to much larger numbers by the year 2000 (e.g., 1,873 instead of 208 breeding pairs/10,000 km^2^). Most (2/3) of the poisoned dead birds located in the English study area of reintroduced kites [21] were deliberately poisoned, whereas the remaining had secondary poisoning from rodenticides. Reduction of this illegal poisoning could make a large difference for recovering red kite populations in the UK. Reduction of lead shot exposure for populations near hunting estates would also increase the recovery rate, although to a smaller magnitude.

Similar to the Welsh population we modeled, red kite populations in Mallorca, Spain continued to grow by at least 8.2%/yr despite high poisoning rates (poison was 43% of mortality for yearlings and 76% for older birds [8], or 53% overall of radio-tagged birds [75]). Another red kite study with a growing population in Scotland reported 40% of the kites died mainly from illegal poisoning [9], which is similar to the 46% we modeled based on the study of the reintroduced population in England [21] (introduced in Chilterns, Midland, Yorkshire, Northumbria). The Welsh population is in an area with poorer habitat than other red kite populations in Europe, resulting in lower fecundity and lower maximum possible growth rate relative to other areas [8–9, 40]. Therefore, changes in survival from poisoning are expected to have a larger effect in Wales than other areas. Nonetheless, probabilities of extinction or a return to the very low bird numbers observed in the 1940s were low for the Welsh population after it reached 50 pairs, even with poison and lead shot exposure at the levels modeled. However, the situation could change quickly and the population could start to decline if poisoning rates (in addition to lead shot ingestion) increase even by a small amount (e.g., with an increase of 10%, resulting in 56% of deaths from poison using our model).

### Sensitivity Analysis

Our sensitivity analysis of fecundity effects indicates that decreased hatching success or other measures of fecundity other than chick survival for the grey partridge can reduce the steady-state population size further (by an additional 6 to 12% when fecundity is reduced by 5 and 10%, respectively) and increase quasi-extinction probabilities (from 4.5% to 8.5%). For raptors, reduced fecundity has a very small effect on the common buzzard, but it slows the annual growth of the red kite population by 25% on a relative basis (i.e., from 4 to 3%/yr). More field studies comparing these uncertain measures in areas with high and low densities of lead shot are needed to evaluate if poisons and lead shot are actually reducing reproduction and accelerating the adverse effects of the chemical stressors.

The scenarios in this paper only should be considered “what if” examples, with much uncertainty in the results because (1) fecundity effects are uncertain as discussed above, (2) probability of discovery of poisoned birds may be higher than for birds dying of other causes, (3) compensatory reproduction or mortality is not considered, and (4) immigration and emigration are not explicitly modeled. The second factor (probability of poisoned bird discovery) may bias the model’s estimation of adverse effects high, unless deaths by accidents near human activity areas are overestimated [50], resulting in an underestimate of other mortality factors. The third factor (compensatory mechanisms), which is important for waterfowl (e.g., hunters more often shoot lead-poisoned birds, [76]), may also bias adverse effects high. The last factor, migration, may bias the estimation of adverse effects low if poisoned dispersers do not die on the monitored study area but die elsewhere, if these dispersers reduce reproduction of off-site populations, or if the population modeled is a “sink”, thus attracting individuals from other healthy populations as territories open up from chemical-related deaths [77]. Also, the model results should not be extrapolated to other areas because Europe is very heterogeneous with different hunting systems, habitat conditions, and poison usage patterns.

Of high importance, more documentation of the percentage of total annual mortality proximally and ultimately caused by ingested shot or poisons is needed, because the population models are most sensitive to this input parameter. As discussed in the *Results* section, other model parameters also have uncertainty (θ, λ_max_, baseline survival, fertility). Within the range of their uncertainty, however, they have much smaller effects on the outcome of the stochastic model than the high among-study-area uncertainty in the magnitude of the annual mortality attributed to lead shot or poison ingestion. Additionally, if concentrations of lead in tissue are used to determine the potential ultimate percentage of mortality due to lead shot ingestion (as we did for raptors), one should consider whether or not the percentage might be overestimated because it might include lead poisoning from lead bullets or non-lead-shot sources. For example, lead bullets, instead of lead shot, were estimated to have caused 5.1% annual mortality in California condors (*Gymnogyps californianus*) [78] and are the main lead source for golden eagles poisoned by ammunition in the Swiss Alps [79]. Leaded gasoline appeared to poison European kestrels (*Falco tinnunculus*) [80]. Isotopic ratios can help distinguish among these various lead sources [68]. For example, based on isotopic ratios that match lead shot, the lead in liver tissues of the red kites we used for mortality estimates was compatible with lead shot [21].

We encourage similar population-level analyses of effects of more than one type of poison or stressor be conducted on other species of concern that are sensitive to chemicals in the environment (e.g., Egyptian and bearded vultures [*Neophron percnopterus* and *Gypaetus barbatus*], white-tailed eagles [*Haliaeetus albicilla*]). Often, only one chemical stressor is evaluated in population models [8–9]. Although our analysis focused on three terrestrial species that feed on non-wetland, terrestrial food sources, our multi-stressor approach could be applied to raptors and waterfowl feeding in aquatic systems, which typically have higher percentages of deaths attributable to lead shot ingestion than terrestrial species [4, 20].

In conclusion, for our modeled populations representing three terrestrial bird species, ingestion of lead shot, pesticides, and bait poisons for the modeled scenarios showed that, although the populations have low probability of quasi-extinction, the poisons can reduce population sizes and substantially slow their recovery rates. Because the outcome is unknown for other species or different locations, more studies and similar modeling efforts will be needed to evaluate effects of multiple stressors and to quantify the variability and uncertainty in results among study areas and species. Our results support that multiple chemical stressors such as lead shot and poison ingestion can have adverse effects on terrestrial bird populations. In particular, removal of illegal and secondary poisons in baits can have a large positive impact on recovering populations of raptors such as the red kite that frequently feed on carrion. Future work should evaluate additional mortality factors to understand effects of multiple stressors on the population dynamics of species.

## Supporting Information

S1 Fig Modeled deterministic density-dependent relationships for grey partridge. Relationships are between grey partridge continental population density (N/km^2^, where N = number) and three factors: (1) annual per capita growth rate, r (r = ln λ, where λ = annual population growth factor), (2) natural log of spring per capita growth rate [ln(λ_spring_)], and (3) autumn-winter survival (σ^aw^). Equations defined in De Leo et al. [10] for the survival and spring growth curves are: σ^aw^ = e^(-0.55–0.0021·N)^, λ_spring_ = e^(1.172–0.0073·N)^. The De Leo et al. [10] model is based on densities, rather than population sizes because populations are patchy and total population size for continental Europe is unknown.
(TIF)

S2 Fig Modeled density-dependent relationships for common buzzard and red kite. Relationship between population size and per capita growth rate, r (= ln λ, where λ = annual population growth factor), for common buzzard and red kite is shown in (a). Relationship between fecundity (F#, where # is age class) and survival (S#) is also shown for common buzzard (b) and red kite (c). The vital rates in (b) and (c) produce the theta-logistic curves in (a).
(TIF)

S3 Fig Relationship between per capita growth rate, r, and density of common snail kite breeding pairs in Florida, USA [56]. r = lnλ, where λ is annual population growth factor. The shape of the curve is described by theta (θ) in a theta-logistic equation [r = r_max_(1 –(N/K)^θ^] and is equal to 5.58 (where N = population size, K = carrying capacity, r_max_ = r at N = 0). This high θ indicates r does not decrease much until density is close to K, the steady-state population size (i.e., where the curve crosses the r = 0 line).
(TIF)
