# Supplementary figures and images for: Can Ingestion of Lead Shot and Poisons Change Population Trends of Three European Birds: Grey Partridge, Common Buzzard, and Red Kite?
Source: PLoS One. 2016 Jan 22;11(1):e0147189. doi: 10.1371/journal.pone.0147189 (PMC4723309; doi:10.1371/journal.pone.0147189)

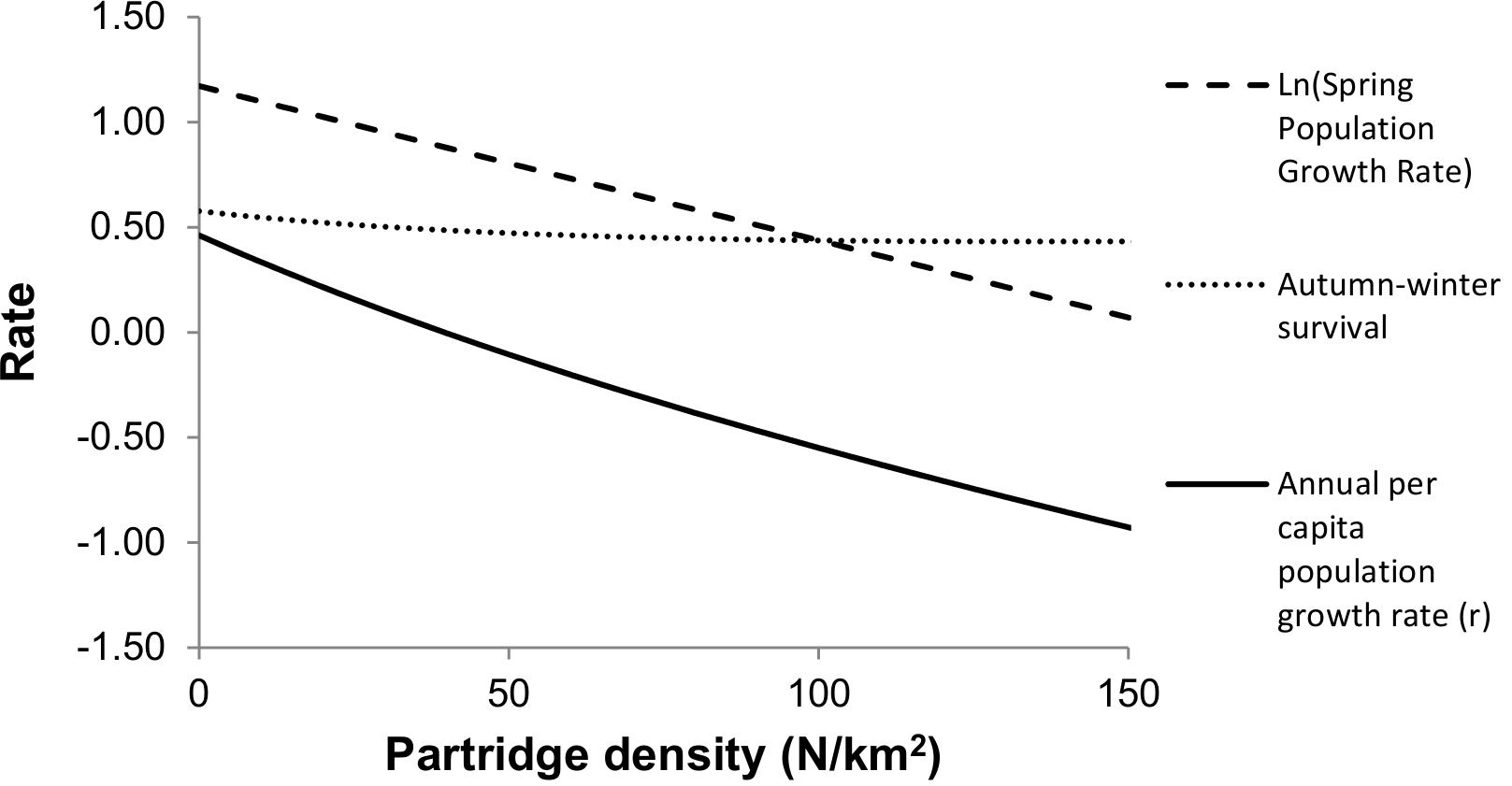

Supplement: S1 Fig — Relationships are between grey partridge continental population density (N/km2, where N = number) and three factors: (1) annual per capita growth rate, r (r = ln λ, where λ = annual population growth factor), (2) natural log of spring per capita growth rate [ln(λspring)], and (3) autumn-winter survival (σaw). Equations defined in De Leo et al. [10] for the survival and spring growth curves are: σaw = e(-0.55–0.0021·N), λspring = e(1.172–0.0073·N). The De Leo et al. [10] model is based on densities, rather than population sizes because populations are patchy and total population size for continental Europe is unknown. (TIF) [file pone.0147189.s001.tif]

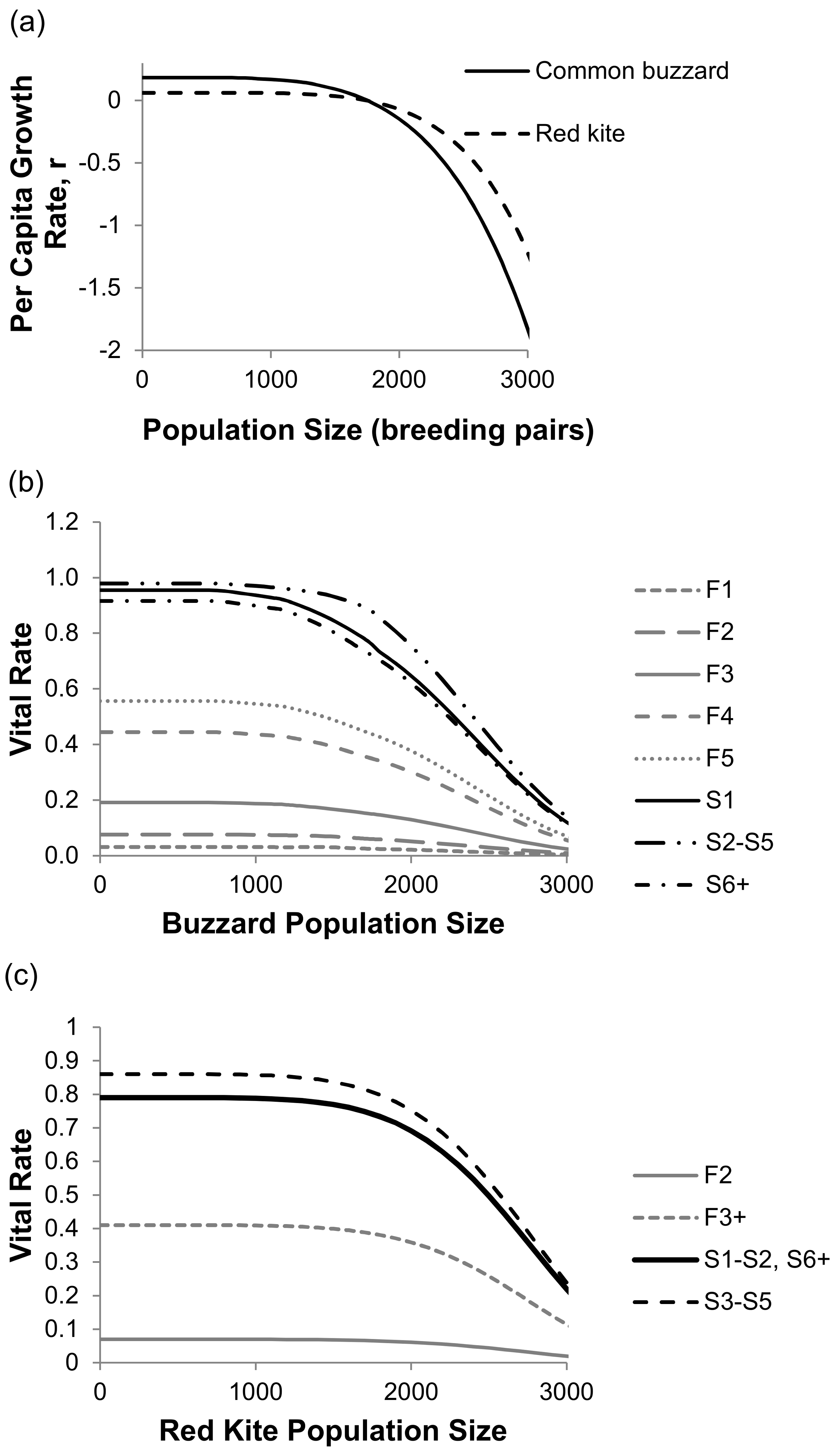

Supplement: S2 Fig — Relationship between population size and per capita growth rate, r (= ln λ, where λ = annual population growth factor), for common buzzard and red kite is shown in (a). Relationship between fecundity (F#, where # is age class) and survival (S#) is also shown for common buzzard (b) and red kite (c). The vital rates in (b) and (c) produce the theta-logistic curves in (a). (TIF) [file pone.0147189.s002.tif]

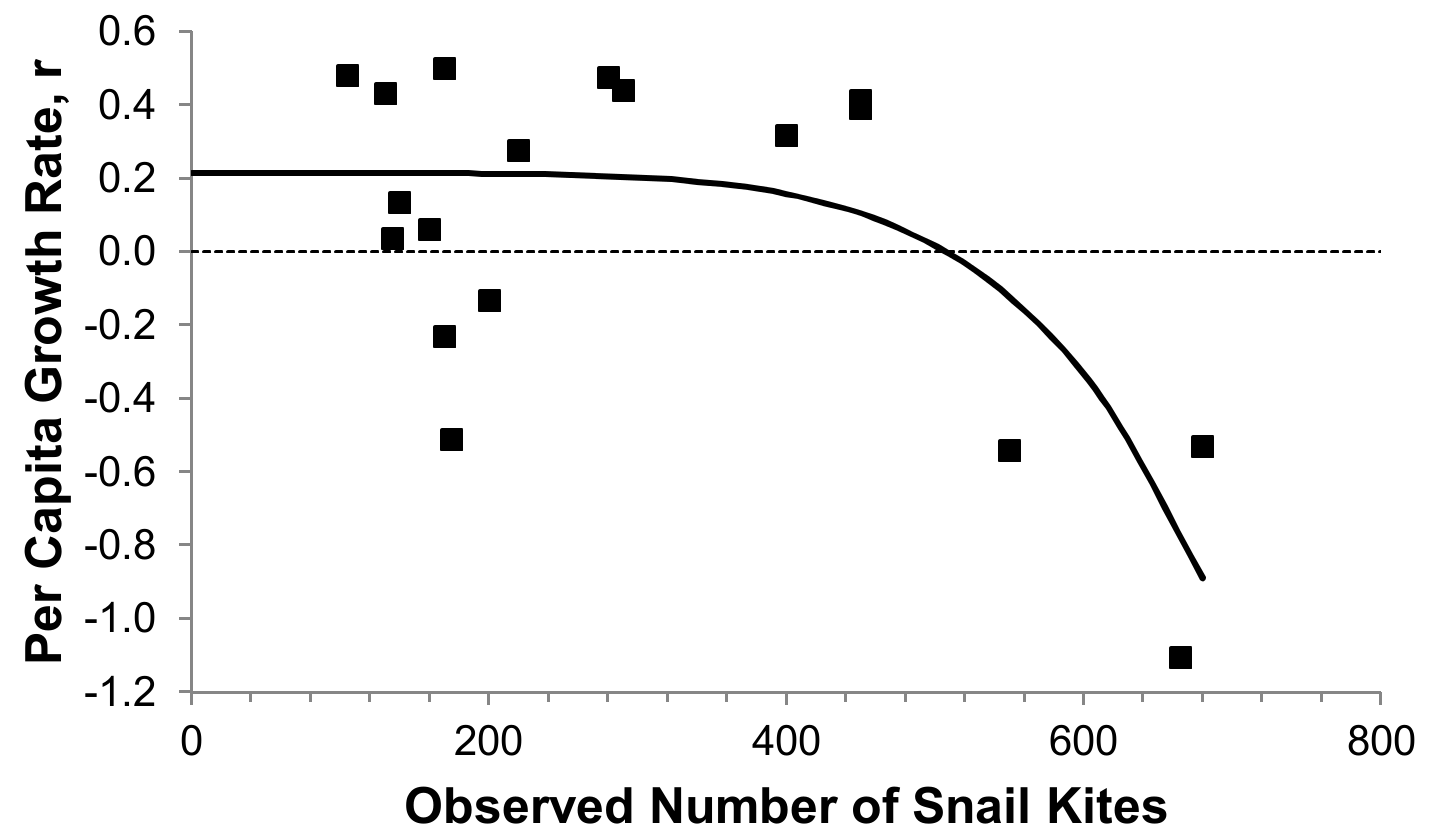

Supplement: S3 Fig — r = lnλ, where λ is annual population growth factor. The shape of the curve is described by theta (θ) in a theta-logistic equation [r = rmax(1 –(N/K)θ] and is equal to 5.58 (where N = population size, K = carrying capacity, rmax = r at N = 0). This high θ indicates r does not decrease much until density is close to K, the steady-state population size (i.e., where the curve crosses the r = 0 line). (TIF) [file pone.0147189.s003.tif]
